# Genetic analysis of multifocal superficial urothelial cancers by array-based comparative genomic hybridisation

**DOI:** 10.1038/sj.bjc.6603850

**Published:** 2007-06-19

**Authors:** H Kawanishi, T Takahashi, M Ito, Y Matsui, J Watanabe, N Ito, T Kamoto, T Kadowaki, G Tsujimoto, I Imoto, J Inazawa, H Nishiyama, O Ogawa

**Affiliations:** 1 Department of Urology, Graduate School of Medicine, Kyoto University, 54 Shogoin Kawahara-cho, Sakyo-ku, Kyoto 606-8507, Japan; 2 Department of Genomic Drug Discovery Science, Graduate School of Pharmaceutical Sciences, Kyoto University, 46-29 Yoshida-Shimo-Adachi-cho, Sakyo-ku, Kyoto 606-8501, Japan; 3 Eisai Co. Ltd., 5-1-3 Tokodai, Tsukuba, Ibaraki 300-2635, Japan; 4 Department of Molecular Cytogenetics, Medical Research Institute and School of Biomedical Science, Tokyo Medical and Dental University, 1-5-45 Yushima, Bunkyo-ku, Tokyo 113-8510, Japan; 5 Core Research for Evolutional Science and Technology of Japan Science and Technology Corporation, 4-1-8 Honcho Kawaguchi, Saitama 332-0012, Japan; 6 21st Century Center of Excellence (COE) Program for Molecular Destruction and Reconstitution of Tooth and Bone, 1-5-45 Yushima, Bunkyo-ku, Tokyo 113-8510, Japan

**Keywords:** multifocal low-grade urothelial cancer, array-CGH, genetic alterations

## Abstract

The purpose of this study was to investigate the accumulation of genetic alterations during metachronous and/or synchronous development of multifocal low-grade superficial urothelial tumours in the same patient, by using array-based comparative genomic hybridisation (array-CGH) and FGFR mutation analysis. We analysed 24 tumours (pTa-1 G1-2) from five patients. We had previously identified a clonal relationship among the tumours of each patient by microsatellite analysis. This time, unsupervised hierarchical cluster analysis revealed that the tumours from each patient were clustered together independently of the tumours from the other patients. All of the tumours from a single patient showed a set of 2–7 identical regional or whole-arm chromosomal changes. In addition, several individual alterations were also found. Cladistic diagrams revealed that the accumulation of genetic alterations could not be explained by a linear model, and the existence of a hypothetical precursor cell was assumed in four patients. In some cases, FGFR mutation seemed to occur later during multifocal tumour development. Taken together, these findings suggest that low-grade superficial urothelial tumours accumulate minor genetic alterations during multifocal development, although these tumours are genetically stable.

It is now commonly accepted that solid primary cancers, including urothelial tumours and cancer of the colon, breast, and lung, arise due to a multistep process involving the accumulation of genetic alterations (Hittelman, 2001; Almadori *et al*, 2004). It is generally difficult to follow the chronology of genetic alterations that accompanies growth of a human tumour due to therapeutic intervention. Therefore, the postulated steps in the process of carcinogenesis are almost invariably based on retrospective comparison of the genomic alterations in tumours obtained from different patients that are of different stages and grades. Early steps are therefore defined as genetic alterations that are found in tumours of all grades and at all stages, whereas late steps are only detected in the tumours of later stage and higher grade. Simultaneous or metachronous development of multifocal urothelial tumours is a well-known characteristic of this type of cancer. Urothelial tumours are most often (70%) superficial cancers that can be treated endoscopically, but 60–80% of patients suffer from at least one episode of recurrence after initial treatment. Due to these unique properties, urothelial tumours provide a good model for the clonal analysis and chronological tracing of genetic alterations in human cancer.

There are two theories regarding the multifocal nature of urothelial cancer, which are known as ‘field effect’ and ‘monoclonality’ (Knowles, 2006). Molecular genetic studies support the monoclonality theory and indicate that tumour cells spread to multiple sites by intraepithelial or intraluminal seeding (Sidransky *et al*, 1992; Habuchi *et al*, 1993; Takahashi *et al*, 1998; Dalbagni *et al*, 2001), while other studies have shown that field effect underlies some multifocal urothelial tumours (Paiss *et al*, 2002; Jones *et al*, 2005). An understanding of the mechanism leading to accumulation of genetic alterations during multifocal tumour development may provide new prospects for both the early detection and prevention of the recurrence of urothelial cancer.

The existence of two distinct groups of tumours with different clinical features is also a striking feature of urothelial cancer. More than 70% of tumours are low-grade superficial cancers at diagnosis. These commonly recur, but progression to muscle invasion is relatively infrequent (10–20%) and the prognosis is usually good. In contrast, about 20% of tumours show muscle invasion at diagnosis, and these have a poor prognosis with <50% survival after 5 years. Furthermore, these tumour types are also distinct at the molecular level. Over 70% of the low-grade superficial cancers have *FGFR3* mutations versus only 10–20% of invasive cancers, strongly suggesting that activation of *FGFR3* is one of the key genetic events underlying the development of low-grade superficial urothelial cancer (Wu, 2005). Recent array-based comparative genomic hybridisation (array-CGH) and SNP array analyses have shown that DNA copy number changes are more frequent in invasive cancer than in low-grade superficial tumours (Primdahl *et al*, 2002; Blaveri *et al*, 2005).

These two distinct types of cancer can both be multifocal, but the extent or pattern of clonal evolution during development seems to differ between them. Simon *et al* (2001) conducted a cytological analysis of multifocal invasive bladder cancer by CGH. They reported that all the tumours of a single patient were derived from a common progenitor cell and subsequently acquired various additional genetic alterations. In the case of low-grade superficial tumours, synchronous or metachronous lesions obtained from the same patient are also generally reported to show a striking degree of identity in their genetic alterations (Takahashi *et al*, 1998; Zhao *et al*, 1999). However, previous studies of low-grade superficial tumours focussed on a few specific genetic changes, and did not investigate chromosomal gains and losses throughout the entire tumour genome. By examining more samples from each patient using array-CGH with a higher resolution, we should be able to draw a more detailed genetic tree and pedigree for multifocal urothelial tumours and trace the genetic alterations that accumulate during their development.

In this study, we used the MCG Cancer Array-800 with 800 target bacterial artificial chromosome (BAC) clone DNAs and FGFR mutation analysis to examine possible genetic divergence and the presence of a genetic hierarchy during the development and recurrence of urothelial cancer.

## MATERIALS AND METHODS

### Patients and tumour samples

Twenty-four physically distinct urothelial tumours of the bladder, ureter, and renal pelvis that were obtained from five patients were included in this study. The clinical and pathological data are summarised in Table 1. Most of the tumour specimens were obtained by transurethral resection (TUR). Samples were snap-frozen and stored at −80°C until the extraction of DNA. An adjacent or deeper portion of the same tumour specimen was used for the histopathological examination. In the case of renal pelvic or ureteral tumours, tissue specimens were collected under direct vision and subjected to histopathological analysis along with DNA extraction. The location of each tumour was recorded. If a tumour recurred at the same location less than 3 months after TUR, the lesion was excluded because there was a high probability of incomplete resection. Also excluded were tumour specimens that contained more than 20% normal interstitial cells on haematoxylin and eosin staining. All of the tumours were low-grade (grade 1–2 by 1998 WHO criteria) superficial cancers. Normal reference DNA was obtained from peripheral blood samples in each patient. Tumour DNA and reference DNA samples were prepared by proteinase K digestion and phenol/chloroform extraction.

These tumours were analysed in our previous LOH study and a clonal relationship was already defined (Takahashi *et al*, 1998). Using microsatellite markers, we examined genetic alterations at 20 loci on eight chromosome arms (2q, 4p, 4q, 8p, 9p, 9q, 11p, and 17p). Completely identical LOH patterns were detected in three patients (Patients 1, 2, and 31), while a mixture of discordant patterns together with concordant patterns were observed in two patients (Patients 16 and 21). Written informed consent was obtained from each patient before surgery according to the ethical guidelines of our university.

### Array-CGH analysis

Array-CGH analysis was performed with the MCG Cancer Array-800, which consists of ∼800 BACs harbouring 800 known cancer-related genes, and is intended for diagnosis of cancer-specific copy number aberrations (Sonoda *et al*, 2004). Array-CGH experiments were carried out as described previously (Takada *et al*, 2005) with minor modifications. Briefly, *Dpn*II-restricted test DNA (tumour DNA) and reference DNA (peripheral blood genomic DNA) were labelled by random priming with Cy3- and Cy5-dCTP (Amersham Biosciences, Tokyo, Japan), precipitated with ethanol in the presence of Cot-1 DNA, redissolved in a hybridisation mixture (50% formamide, 10% dextran sulphate, 2 × standard saline citrate (SSC), and 4% sodium dodecyl sulphate, pH 7), and denatured at 75°C for 8 min. After incubation at 42°C for 30 min, each mixture was applied to array slides and incubated at 50°C for 10 min, 46°C for 10 min, and 42°C for 60 h in a hybridisation machine (Hybrimaster HS-300, Aloka, Tokyo, Japan). Hybridised slides were washed once in a solution of 50% formamide and 2 × SSC (pH 7.0) for 10 min at 50°C, and then in 1 × SSC for 10 min at 42°C. After air-drying, the arrays were scanned with a GenePix 4000B (Axon Instruments, Foster City, CA, USA), and the images thus acquired were analysed with GenePix Pro 4.1 imaging software (Axon Instruments). Fluorescence ratios were normalised so that the mean of the middle third of log 2 ratios across the array was zero. A global threshold of ±0.40 defined gain or loss for all of the BAC clone log 2 ratios. High-level amplification and homozygous deletion were defined by a log 2 ratio of >2 and <−2, respectively.

### Analysis of array-CGH data

Among the 800 BAC clones, 17 spots were omitted from analysis because of collation with the latest genomic data and re-evaluation of clone design. We performed unsupervised hierarchical cluster analysis of log 2 ratios obtained for the target BAC clones to determine whether the prevalence of genomic changes was similar or different. The Ward linkage and cosine coefficient metric were used, and results were assessed with the software program R.

Contiguous alterations were expressed as regional or whole-arm changes by calculating the median log 2 ratio of all BAC clones mapping to each altered region of a chromosome. A threshold of ±0.30 was then applied to the median log 2 ratio to define ‘regional’ gains or losses relative to the reference sample.

### *FGFR3* mutation analysis

Mutations of *FGFR3* were identified by direct sequencing of tumour DNA. Three regions of interest containing the previously identified mutations were amplified by PCR (Jebar *et al*, 2005) using the following primer pairs: for exon 7, 5′-AGTGGCGGTGGTGGTGAGGGAG-3′ and 5′-TGTGCGTCACTGTACACCTTGCAG-3′; for exon 10, 5′-CCTCAACGCCCATGTCTTT-3′ and 5′-GGGAGCCCAGGCCTTTCTTG-3′; and for exon 15, 5′-CCGCAATGTGCTGGTGAC-3′ and 5′-GGCGTCCTACTGGCATGA-3′. PCR amplicons were purified using a PCR purification kit (Qiagen, Hilden, Germany) and then directly sequenced with the same primers that were used for the initial PCR and the ABI Prism 310 genetic analysis system (Applied Biosystems, Warrington, UK).

## RESULTS

### Array-CGH analysis

A file containing the raw CGH array data is available as Supplementary information. Among the 783 BAC clones examined in each of 24 tumours (18 792 in total), gain and loss were observed in 567 (3.0%) and 1299 (6.9%), respectively. The most common alterations of the chromosome arms were 9q loss (79%), 9p loss (75%), 11p loss (67%), 20q gain (50%), 17p loss (46%), and 11q loss (38%). Alterations of these regions were also reported in two previous array-CGH studies of bladder cancer (Veltman *et al*, 2003; Hurst *et al*, 2004). Homozygous deletions were observed at the locus where clones (9p21) containing the *MTAP* gene and *CDKN2B (p16)* gene were mapped in eight tumours of two patients, whereas high-level amplifications were not detected.

Copy number alterations were found in a large fraction of most tumours. The extent of genomic changes was defined as the fraction of the genome altered (FGA), as described by Blaveri *et al* (2005). Each clone was assigned a genomic distance equal to the sum of one-half of the distance between its own centre and that of its two neighbouring clones. The average genomic distance between clones resulting from this calculation was ∼3.5 Mb. Overall, each tumour had an average 9.5% (2.5% gain and 7.0% loss) frequency of alterations (Table 1).

**Figure 1:**
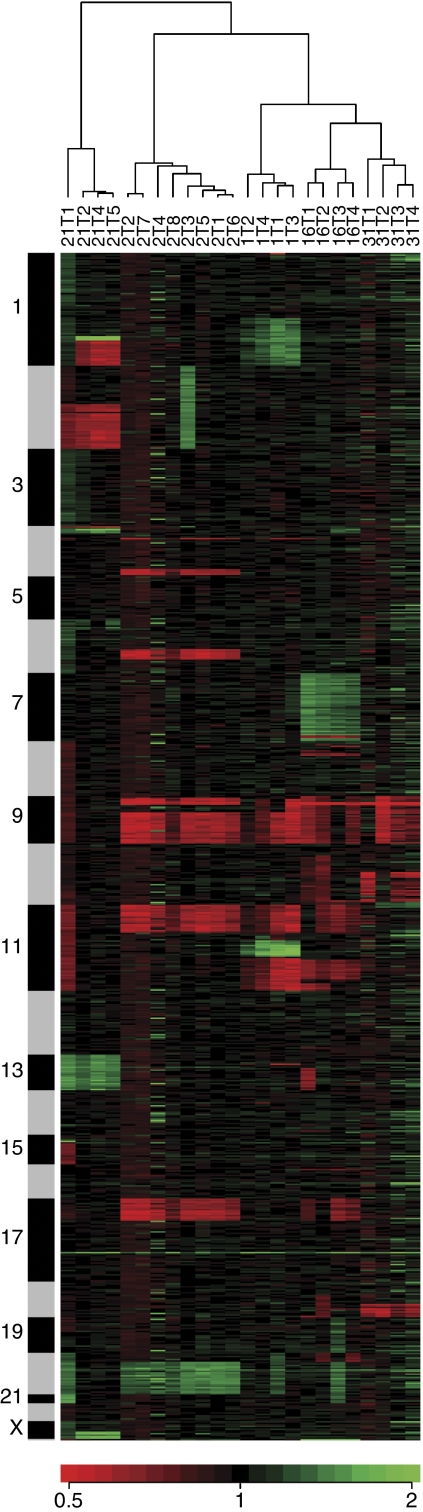
Hierarchical clustering of data for 24 tumours obtained from clones in the MCG Cancer Array-800. The data are presented in a matrix format. Each column corresponds to a single tumour, and each row corresponds to a single clone ordered by mapping position. Gain or loss of a clone is represented by the colour of the cells in the matrix (green indicates gain, black is no change, and red means loss). Colour saturation is proportional to the magnitude of the difference. The sidebars to the left of the matrix format represent a chromosome cluster ordered from chromosome 1 to Y. The horizontal dendrogram shows that the associations between tumours and the length of the branches reflect the extent of similarity between tumours. Note that each tumour from a particular patient is more closely related to other tumours from the same patient than to any tumours from the other patients.

Figure 1 shows the cluster dendrogram for the 24 tumours of the five patients based on the similarity of genetic alterations detected by array-CGH. Tumour pairs from each patient were clustered together. One tumour was related more closely to the other tumours from the same patient than to the tumours from any of the other patients, suggesting that these tumour pairs were clonally related.

**Figure 2:**
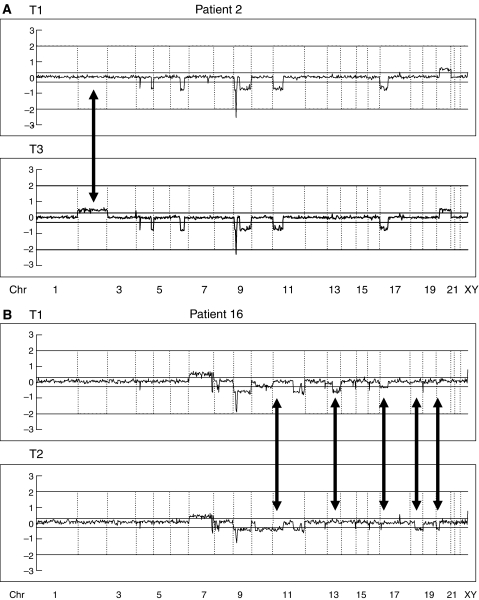
Graphical representation of array-CGH analysis of tumours from Patients 2 and 16 (whole genome). Vertical lines indicate the boundaries of the chromosomes. (**A**) In Patient 2, a concordant pattern was found in 4q32-35, 6q15-22, 9p21-24, 9q, 11p, 17p (loss) and 20q (gain). A later tumour (T3) had additional genetic alterations (2p, 2q (gain)). (**B**) In Patient 16, several discordant patterns were found in 11p, 13q22-tel, 17p, 18q, and 20p. Average log 2 ratios were plotted for all clones at the chromosome positions. Thresholds for gain or loss are shown within log 2 ratios of 0.3 and −0.3, respectively. Arrows indicate the differences of the pattern of chromosomal aberrations between tumour pairs. T=tumour; Chr=chromosome.

All of the tumours derived from a single patient showed a set of 2–7 identical regional changes or whole-arm changes, although several additional individual alterations were also found. Representative array-CGH profiles are presented in Figure 2.

### *FGFR3* mutation analysis

Mutations of *FGFR3* are strongly associated with a low tumour grade and stage, with up to 60–70% of low-grade pTa tumours showing these mutations (Billerey *et al*, 2001; van Rhijn *et al*, 2001; Jebar *et al*, 2005). Therefore, in addition to chromosomal alterations, *FGFR3* mutations are used as a clonal marker. A total of 23 *FGFR3* mutations were found in the 24 tumours (Table 1). In three patients (Patients 2, 16, and 31), all of their tumours had the S249C mutation. In Patient 21, all tumours had the Y375C mutation. Such results suggested that the multifocal tumours of these four patients were clonally related. In Patient 1, however, tumours 2–4 had the Y375C mutation, while tumour 1 was WT FGFR3. This suggests that FGFR mutation may occur later during multifocal tumour development in some cases.

### Cladistic diagrams of the accumulation of genetic alterations during multifocal tumour development

**Figure 3:**
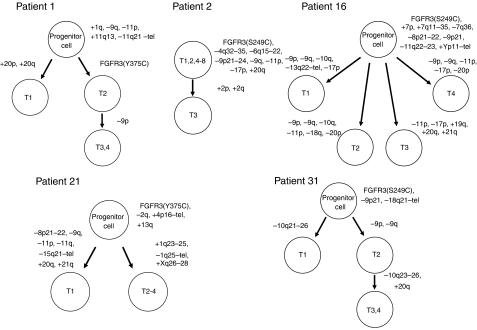
Evolutionary genetic tree depicting the relationship between the multiple tumours of each patient.

Based on the results obtained with respect to array-CGH and *FGFR3* mutations, possible schematic pathways of the genetic alterations that occur during the development of multifocal low-grade superficial urothelial tumours are presented in Figure 3. Using the theory of accumulation of genetic changes during tumour development, we developed cytogenetic pedigrees that reflected the accumulation of chromosomal aberrations in each case. An apparent pattern of accumulation of genetic alterations was detected in three patients (Patients 1, 2, and 31). Patient 2 had tumours that were genetically stable because seven (T1, 2, 4–8) out of eight tumours had the same pattern of chromosomal aberrations, and only one tumour (T3) showed additional alterations. In the remaining four patients (Patients 1, 16, 21, and 31), the accumulation of genetic alterations could not be explained by a linear model, so a hypothetical precursor cell was assumed. In particular, Patient 16 had tumours with diverse additional chromosomal aberrations that were presumably acquired from a hypothetical precursor cell. In the light of these results, the possibility was suggested that undetected tumour cells already existed at the time of first presentation in Patients 1, 16, and 21.

### Comparison of array-CGH and PCR-based microsatellite analysis

Both the microsatellite data and the CGH array results are available as Supplementary information. The loci of the 20 microsatellite markers used in the previous study did not correspond exactly with the target DNAs for array-CGH. Among the microsatellite markers used in the previous study, 12 markers for which the distance to the nearest spot on the array was less than 1 Mb were selected to compare the results of the two methods. Of 184 informative microsatellite analyses using the 12 markers, 107 showed LOH. Overall, 124 out of 184 informative microsatellite analyses (67.3%) matched the results of array-CGH. Among the 107 sites of LOH, 61 (57.0%) were detected as gains or losses by array-CGH. Thus, the results of the array-CGH and microsatellite analyses did not match closely, probably because the loci of most microsatellite markers did not correspond with the spots on the array. In addition, the different results have been related to differences of the assay method because microsatellite analysis involves amplification, while array-CGH does not.

## DISCUSSION

Chronological analyses of multifocal tumours have suggested that the clinical presentation of each lesion may not necessarily be correlated with the order in which genetic alterations occur or with biological tumour development (Habuchi, 2005). In the present study, cladistic diagrams revealed that the genetic aberrations of individual tumours could not be explained by a linear model, and the existence of a hypothetical precursor cell was assumed in four of our five patients. In addition, we found that the order in which tumours presented clinically was not the same as the order of development in Patient 2. Interestingly, van Tilborg *et al* (2000) reported similar findings to these in an LOH-based analysis of multifocal bladder cancer. A tumour with additional genetic alterations may develop earlier than a tumour without such alterations, even though both lesions are derived from the same precursor cell. This finding may provide interesting information for the development of appropriate treatments and surgical strategies. According to a recent meta-analysis, a single instillation of a chemotherapy agent immediately after TUR significantly reduces the recurrence rate during the first and second years in patients with stage Ta/T1 bladder cancer (Oosterlinck *et al*, 2005). This effect has been explained by the prevention of tumour seeding at the time of TUR. However, there is some evidence that relapse occurs at a similar rate in both the intravesical chemotherapy and observation groups after this early period (Oosterlinck *et al*, 2005). This may be explained by the concept that some patients with multifocal low-grade tumours already have undetected tumour cells at various sites at the time of first presentation due to intraluminal seeding or intraepithelial spread.

Studies based on molecular genetics have suggested a monoclonal origin for multifocal urothelial tumours, while other studies have clearly shown an independent origin for some multifocal cancers. Thus, both of these mechanisms appear to operate during multifocal tumour development. Most of the previous studies investigated advanced and high-grade cancers, while few studies have focussed on low-grade superficial tumours. Several reports have indicated that superficial multifocal bladder tumours are most likely to be of monoclonal origin (Takahashi *et al*, 1998; Li and Cannizzaro, 1999; Louhelainen *et al*, 2000). For this study, we chose tumours that appeared to be clonal from the results of our previous LOH study. Cluster analysis showed that each tumour was more closely related to the other tumours of the same patient than to any of the tumours from the other patients (Figure 1), and cladistic diagrams revealed how these tumours evolved (Figure 3). Our results suggested that the tumours of most patients were clonally related.

Progression of genomic instability that correlates with the stage of a tumour has been described (Koed *et al*, 2005), while a study indicated that certain chromosomal areas are frequently altered during the progression of bladder cancer (Primdahl *et al*, 2002). When Koed *et al* (2005) studied at least two tumours of different stages from the same patient, they found that allelic imbalances were more common in later stage (T2-4) tumours than in earlier stage (Ta-1) tumours. In the present study, low-grade superficial tumours (22 were pTa and 2 were pT1) had a 9.5% average frequency of alterations. This was consistent with the findings of Blaveri *et al* (2005), who showed that low grade pTa tumours had a much lower FGA (median: 8%) than pT1 tumours (27%) or those with muscle invasion (18%). Additional genetic alterations occur relatively infrequently, and the overall FGA does not change dramatically during multifocal tumour development (Table 1). Although low-grade urothelial tumours accumulate minor genetic alterations during multifocal development, the extent of such alterations is small relative to the entire genome and these lesions are genetically stable. Furthermore, the majority of the additional genetic alterations does not affect the biological behaviour of these tumours and might not confer any survival advantage on the tumour cells. Our data are consistent with the view that deletions involving chromosome 9 and mutations of *FGFR3* occur early in the development of low-grade papillary urothelial tumours (Knowles, 2006). Interestingly, these alterations sometimes occur later during multifocal tumour development (e.g. Patient 1), so the patterns of accumulation of genetic alterations seem to vary among tumour clones.

The array-CGH method has made a significant impact on cancer cytogenetics. Quantitative measurement of DNA copy numbers across the genome has revealed various oncogenes (Tanami *et al*, 2005) or tumour suppressor genes (Takada *et al*, 2005, 2006) that had not been detected by earlier techniques. In addition, the higher resolution of array-CGH has allowed precise mapping of the boundaries of the gained and lost regions, thereby helping to elucidate the clonal relationship between multifocal tumours (Shelley Hwang *et al*, 2004; Wa *et al*, 2005). In conclusion, we investigated the genetic alterations that occur during the development of multifocal low-grade superficial urothelial tumours by using array-CGH, and assessed the mechanism underlying the heterotopic recurrence of urothelial cancer. Our results indicated that array-CGH is a powerful tool for the genetic analysis of multifocal urothelial tumours.

## Figures and Tables

**Table 1:** Characteristic of 24 urothelial tumours of five patients

**Patient**	**Tumour**	**Date**	**Site^a^**	**Grade**	**pT**	**FGA b(%)**	***FGFR3* status**
1	1	November 1994	B5	2	a	8.2	WT
	2	May 1995	B1	2	a	2.4	Y375C
	3	August 1996	B3	1	a	12.4	Y375C
	4	October 1997	B5	1	a	3.6	Y375C
2	1	May 1997	B2	1	a	10.2	S249C
	2	August 1997	B3	1>2	a	9.3	S249C
	3	October 1997	B2	1>2	a	17.8	S249C
	4	—	B2	1>2	a	10.2	S249C
	5	—	B4	1>2	a	10.2	S249C
	6	November 1997	P	1,2	a	9.7	S249C
	7	—	P	1,2	a	9.4	S249C
	8	—	P	1,2	a	4.8	S249C
16	1	January 1997	U	2	a	13.9	S249C
	2	August 1997	B1	2	a	11.7	S249C
	3	—	B2	2	a	8.9	S249C
	4	—	B2	2	a	7.7	S249C
21	1	May 1997	B1	2	1a	15.4	Y375C
	2	October 1997	B2	2	1a	10.8	Y375C
	4	January 1998	B2	2>1	a	12.4	Y375C
	5	—	B2	2>1	a	12.3	Y375C
31	1	January 1998	B1	2	a	5.2	S249C
	2	—	B1	2	a	5.7	S249C
	3	—	B2	2	a	8.7	S249C
	4	—	B2	2	a	8.0	S249C
							

a U=ureteral tumour; P=renal pelvic tumour; B=bladder tumour (the locations of the bladder tumour were as follows: B1=trigone; B2=posterior wall; B3=right wall; B4=left wall; B5=anterior wall).

b Fraction of genome altered.
